# Rare Cause of Abdominal Pain in an Adolescent Patient: Splenic Infarction in Absence of Causative Underlying Hematologic Disorder

**DOI:** 10.7759/cureus.9176

**Published:** 2020-07-14

**Authors:** Deborah Shimshoni, Chrissy Vandillen

**Affiliations:** 1 Medicne, University of Central Florida College of Medicine, Orlando, USA; 2 Emergency Medicine, St. Cloud Regional Medical Center, St. Cloud, USA

**Keywords:** splenic infarcts, child and adolescent, pediatric imaging, emergency medical service, splenic torsion

## Abstract

Splenic infarction is a rare medical condition that usually occurs in the setting of hematologic disorders. It is rarely seen in previously healthy adolescents. A wandering spleen increases the risk of splenic infarct due to risk of torsion and is more commonly seen in pregnancy due to increased elasticity of connective tissue. Wandering spleen may also be associated with diseases, such as Ehlers-Danlos, and should be suspected in the patient with possible underlying connective tissue dysfunction. Although rare, splenic infarction must be on the differential for unremitting upper epigastric pain, fever, and vomiting, particularly when patient medical history suggests connective tissue dysfunction. This case discusses the course of a pediatric patient with abdominal pain with complex medical history found to have splenic infarction secondary to torsion of a wandering spleen initially discovered on emergency CT imaging. Although rarely indicated in pediatric patients with abdominal pain, lower threshold for CT imaging for ruling out emergent etiology resulted in life-saving treatment. This case demonstrates the importance of clinical suspicion for emergency etiology of abdominal pain in pediatric patients with medical history suspicious for connective tissue dysfunction, and therefore lowering the threshold for CT imaging to rule out splenic infarction in these patients.

## Introduction

Splenic infarction is a rare medical condition that is difficult to diagnose in the emergency department. The spleen’s extensive vascularity utilizes 5% of the body’s cardiac output, making it susceptible to emboli and thrombosis from hematological disorders [1]. For this reason, splenic infarction more commonly presents in patients with an underlying hypercoagulable state or hematologic disorder such as Gaucher disease or lymphoma [2]. This etiology is rarely ever involved in a previously healthy adolescent patient presenting with abdominal pain. Even in the patient without a hematologic disorder, the possibility for splenic infarction increases if risks are present for splenic torsion. These indications include splenomegaly or wandering spleen. Wandering spleen is the term used to define a spleen that is not adhered to the peritoneum by usual attachments, but instead is attached primarily by a vascular pedicle [3]. This may lead to torsion about the vascular pedicle, cutting off the spleen's blood supply. Most cases of wandering spleen are observed in women of age 20-40 years and may be attributed to multiparity and subsequent increased laxity of connective tissue [3,4]. Wandering spleen is a rare anatomical finding in children and has been associated with Ehlers-Danlos syndrome due to increased laxity of ligaments [5]. These patients with Ehlers-Danlos associated wandering spleen may eventually succumb to splenic torsion, though such cases are still considered rare and are sparsely available in the current literature [5,6]. This case demonstrates a pediatric patient found to have splenic infarction without underlying hematologic causation, but with a possible connective tissue disorder component. This case highlights when to suspect and investigate the possibility of splenic infarction in patients with abdominal pain, nausea, and vomiting.

## Case presentation

A 16-year-old female presented to a community emergency department with a chief complaint of cramping abdominal pain for two weeks and severe worsening pain with associated nausea and vomiting for two days. She also described sharp pain with inspiration in her left anterior chest. The patient had a medical history that included gastroesophageal reflux disease (GERD), carpal tunnel syndrome, tumor pilomatrixoma of the upper extremity, glaucoma, and splenomegaly of unknown etiology. She denied any recent travel, trauma, or sick contacts. She denied any recent history of sore throat, cough, lightheadedness, leg swelling, diarrhea, hematemesis, hematochezia, or shortness of breath.

She appeared pale with dry mucus membranes and was visibly uncomfortable in pain. Vital signs on exam demonstrated slightly elevated temperature of 99.5 degrees Fahrenheit and tachycardic heart rate of 110 beats per minute. Otherwise, vitals were unremarkable with a blood pressure of 132/75 mmHg, a respiratory rate of 18 breaths per minute, and oxygen saturation of 99% on room air. Physical exam revealed diminished bowel sounds and diffuse tenderness to palpation with maximal tenderness located in the epigastrium and bilateral upper abdominal quadrants. There was significant splenomegaly, otherwise no hepatomegaly, masses, or peritoneal signs.

**Figure 1:**
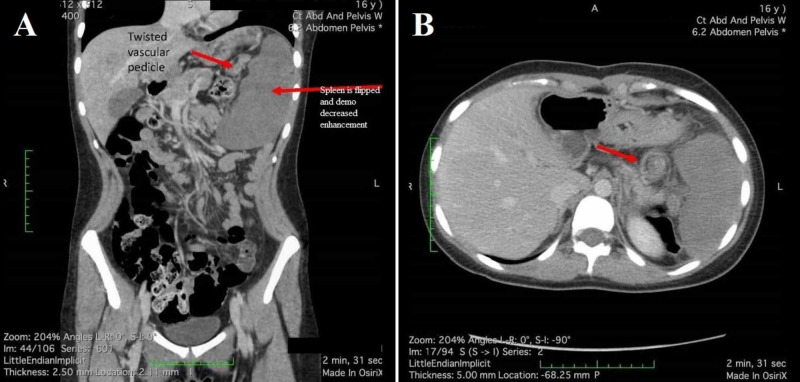
CT with contrast CT with contrast demonstrating splenic infarct secondary to torsion in a 16-year-old female patient. (A) Sagittal view with noted splenic enlargement and twisted vesicular pedicle. (B) Axial view of splenic infarction.

Initial labs revealed an elevated white blood cell count of 23.4 K/µL (reference range 4.1-10.4 K/µL), positive anion gap of 20 (reference range 2-12), but a normal lactic acid dehydrogenase (LDH) of 0.9 mmol/L (reference range 0.4-2 mmol/L). Urine pregnancy test was negative. Due to intractable nausea and continued pain, despite analgesics and anti-nausea medications, CT imaging with contrast was ordered for the abdomen and pelvis. General impression of the CT scan was read by the in-house radiologist as devascularization/infarct of the entire spleen with possible volvulus about the splenic hilum (Figure 1). The patient was immediately transferred via critical care air transport to a tertiary care children’s hospital where emergent surgical intervention revealed non-viable splenic torsion secondary to wandering spleen. Surgical removal of the spleen was successful and the patient had a non-complicated recovery. 

Post splenectomy, the patient was seen by her primary care physician for chronic fatigue, dysmenorrhea, gum bleeding, and joint hypermobility and was subsequently diagnosed with von Willebrand factor deficiency type 1. There is now concern that this patient may have Ehlers-Danlos syndrome. The patient had a wandering spleen, an anatomical finding often associated with Ehlers-Danlos syndrome [5].^ ^This along with the patient’s congenital glaucoma, GERD, and hypermobility increases the suspicion of Ehlers-Danlos diagnosis although final testing for this diagnosis is still pending [6].^ ^

## Discussion

Acute epigastric abdominal pain, nausea, and vomiting with no peritoneal signs, especially in the setting of normal lactate and fever, raises suspicion for gastroenteritis or an infectious etiology in the pediatric patient [7].^ ^CT imaging is not generally suggested for this presentation in order to reduce radiation exposure in the pediatric population. However, exceptions are made if suspicion is high for appendicitis, trauma, pancreatitis, or a palpated mass [7].^ ^If there are continued concerns in patients with abdominal pain such as fever, high white blood cell count, intractable vomiting with continued pain despite medication, CT imaging of the abdomen pelvis should be considered. Pain localized to the left upper quadrant raises suspicion for splenic infarct. In a patient with a complex medical history with a possible connective tissue component, common complaints may reflect a rare underlying etiology.

Splenic infarction may lead to severe consequences if missed on initial emergent exam. Currently, there are limited studies available that attempt to identify the prevalence, etiologies, interventions, and outcomes of splenic infarction in the pediatric population [8]. A massive splenic infarction is described as parenchymal ischemia secondary to vessel occlusion leading to tissue necrosis of at least half of the spleen [9].^ ^For patients below the age of 40 years, etiology is most commonly due to hematologic disease [10]. Other causes include hypercoagulability, trauma, or enlargement secondary to Epstein-Barr viral infection [2,9,10].^ ^Many times, an underlying abnormality is found during the workup for the initial presentation of a splenic infarction [2]. Therefore, even if a patient denies having risk factors of splenic infarction, the differential should still be considered in the previously healthy patient with signs and symptoms that may suggest this diagnosis. A study from 2010 suggests that the most likely symptoms found in order of commonality for splenic infarction include an elevated LDH, localized left abdominal pain or tenderness, white blood cell count >12,000, fever greater than 38˚C, splenomegaly, nausea, and vomiting [2]. Additionally, the presence of a wandering spleen increases the risk of splenic torsion and consequently splenic infarction, a surgical emergency [4]. When the emergency physician is faced with the previously healthy patient with unremitting abdominal pain, splenic infarction should find its way on the differential, particularly in the presence of the aforementioned risk factors or symptoms. This case demonstrates the importance of clinical suspicion to perform a diagnostic procedure for abdominal pain that is not conventionally first line.

## Conclusions

This case demonstrates splenic infarction secondary to splenic torsion in a patient with a chief complaint of nausea, vomiting, and abdominal pain refractory to pharmaceutical intervention found to be tachycardic and febrile on physical exam. Such cases are rare for pediatric patients in the absence of an underlying hematologic disease, and therefore this diagnosis could easily be overlooked due to its vague presentation. This case illustrates the need for emergency physicians to put splenic infarction in the differential diagnosis for patients with intractable vomiting and left upper quadrant abdominal pain. Additionally, this patient’s complicated past medical history supports the possibility of an underlying connective tissue disorder, making splenic involvement more likely. In these patients, emergency physicians should have a lower threshold for CT imaging to rule out an emergent etiology for abdominal pain.
